# Compact meta-spectral image sensor for mobile applications

**DOI:** 10.1515/nanoph-2021-0706

**Published:** 2022-01-14

**Authors:** Jaesoong Lee, Yeonsang Park, Hyochul Kim, Young-Zoon Yoon, Woong Ko, Kideock Bae, Jeong-Yub Lee, Hyuck Choo, Young-Geun Roh

**Affiliations:** Photonic Device Lab., Samsung Advanced Institute of Technology, 130 Samsung-ro, 16678, Suwon, Korea; Department of Physics, Chungnam National University, 99 Daehak-ro, 34314, Daejeon, Korea; Nano Electronics Lab., Samsung Advanced Institute of Technology, 130 Samsung-ro, 16678, Suwon, Korea

**Keywords:** CMOS imager sensor, compact spectral imager, hyperspectral imaging, metasurfaces

## Abstract

We have demonstrated a compact and efficient metasurface-based spectral imager for use in the near-infrared range. The spectral imager was created by fabricating dielectric multilayer filters directly on top of the CMOS image sensor. The transmission wavelength for each spectral channel was selected by embedding a Si nanopost array of appropriate dimensions within the multilayers on the corresponding pixels, and this greatly simplified the fabrication process by avoiding the variation of the multilayer-film thicknesses. The meta-spectral imager shows high efficiency and excellent spectral resolution up to 2.0 nm in the near-infrared region. Using the spectral imager, we were able to measure the broad spectra of LED emission and obtain hyperspectral images from wavelength-mixed images. This approach provides ease of fabrication, miniaturization, low crosstalk, high spectral resolution, and high transmission. Our findings can potentially be used in integrating a compact spectral imager in smartphones for diverse applications.

## 1 Introduction

Optical spectrometers are used in many fields, and applications range from material analysis and astronomy to food chemistry and medical diagnostics. The demand for spectrometers is rapidly growing, but its widespread use is hindered by the size of the spectrometers. As a result, compact spectrometers are highly demanded and reducing instrument sizes has become one of priority fields of study. Various attempts have been made [1] to miniaturize spectrometers such as conventional dispersive mechanism [2], [3], [4], Fourier-transform interferometry (FTI) [5], [6], [7], and the use of detectors with an array of random filters [8], [9], [10], [11] and narrow bandpass filters [12, 13]. The integration of filter arrays with detectors offers an advantage over dispersive and FTI systems because the need for long optical paths and mechanical translation of optical components for high resolution is eliminated. Additionally, integrating filter arrays with detectors such as charge-coupled devices (CCD) and CMOS image sensors (CIS) makes hyperspectral imaging possible by obtaining single-shot two-dimensional images. In particular, integration of narrow bandpass filter array has been presented [14], [15], [16] without the need for post-analysis compared to the random-filter approach. However, a large number of channels for high resolution require a lot of fabrication processes such as etching and deposition because each channel requires a film with different thickness. To address this, combinatorial etch technique was used to fabricate multiple channels [17, 18]. Resonant structures for narrow bandpass filters used in spectrometers were investigated, but most studies are limited to varying the thicknesses of dielectric multilayers to form an optical cavity for different wavelengths and quality factors [19, 20]. This is cumbersome for mass production of devices because it would require an excessive number of dielectric deposition, etching, and photolithography steps, especially at pixel size levels [21].

**Figure 1:**
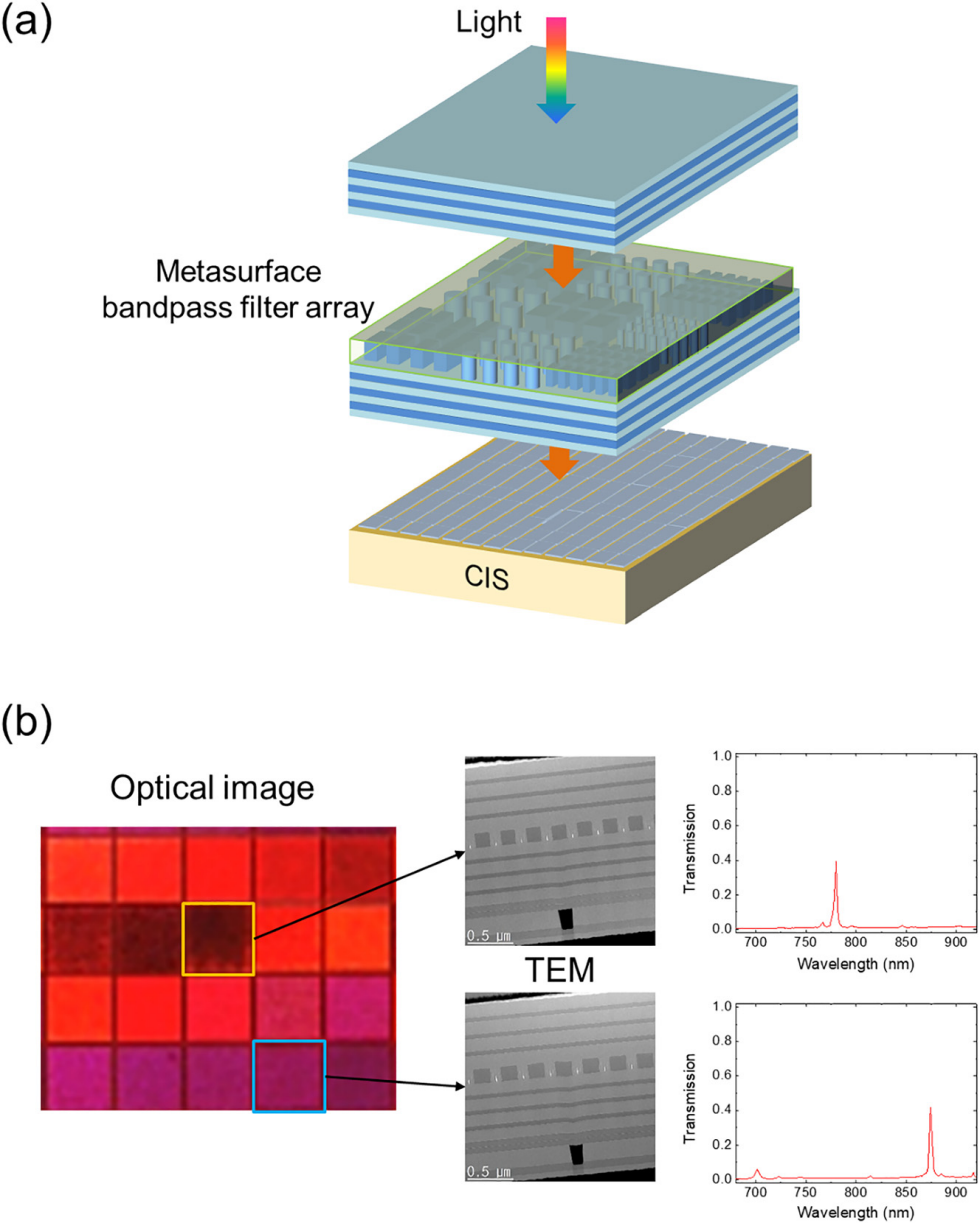
Meta-spectral imager. (a) Schematic of the meta-spectral imager. (b) Optical microscope image of one pixel made of 20 channels (left). Cross-sectional transmission electron microscope (TEM) images of the two channels (center). The measured transmission spectra of the two selected channels (right).

Here, we have fabricated a compact spectral imager by integrating subwavelength nanostructures called metasurface into a bandpass filter array that is directly on top of a CIS as shown in Figure 1. All channels were fabricated by only one lithography process because narrow bandpass filtering was tuned by subwavelength grating structures and not by changing the thickness of layer [22], [23], [24]. This approach simplifies the fabrication and the spectral imager is completely CMOS-compatible. The fabricated device shows high efficiency with narrow bands, low crosstalk with neighboring channels, and high spectral resolution. Using the device, we obtained hyperspectral images from wavelength-mixed images.

## 2 Results

### 2.1 Design of metasurface bandpass filter

**Figure 2:**
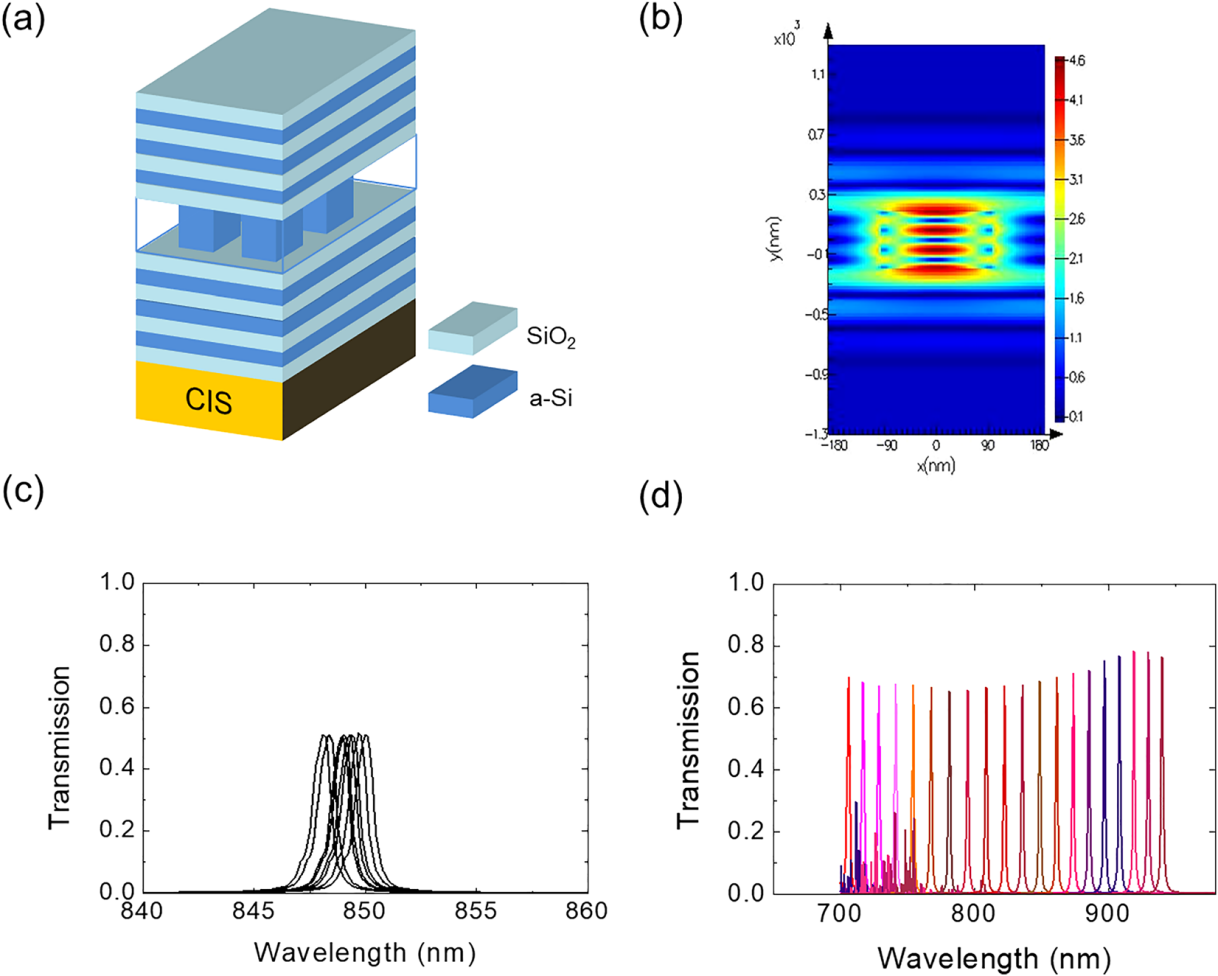
Metasurface bandpass filter array. (a) Schematic of the metasurface bandpass filter. (b) Calculated electric field map confined in the nanopost at the resonant wavelength. (c) Transmission spectra calculated with various etch profiles of posts. (d) Calculated transmission spectra of the nanopost-embedded bandpass filter array.

For the bandpass filter array to cover the 700–950 nm range, we employed nanopost arrays embedded in a small resonator as shown in Figure 2(a). Si nanoposts of equal height varying two-dimensionally in position and size selectively transmit light with certain wavelengths to the CIS. This approach presents great potential for improving various spectrometer characteristics such as a wide angle of incidence and focusing of the transmitted beam. Meanwhile, the compact spectrometer also has to be efficient. Hence, we tried to stabilize the transmitted wavefronts by reducing unwanted light scattering under the top surface of the CMOS imager. A periodic structure is a simple way to meet this demand, thus we performed a rigorous coupled wave analysis (RCWA) using a commercial program (RSoft Photonic Device Tools, DiffractMOD) to design the periodic nanopost structure [25]. In addition to the resonant nanopost array, a few multilayers of dielectric materials were introduced to increase the field confinement inside the nanopost array as shown in Figure 2(b). This resonant cavity structure sharpened the transmission peaks which led to high spectral resolutions.

While the resonator design may be optimal, the real device is still subject to fabrication imperfections such as the surface roughness of each layer and the size difference of patterns. This was accounted for in the simulation by measuring deviations from the design in cross-sectional TEM image and statistically averaging the simulated spectra with feedback from the image. Transmission spectra calculated for various etch profiles of posts are presented in Figure 2(c). After optimizing the design through this process, we calculated the transmission spectra of 20 channels as shown in Figure 2(d) (Supplementary materials). The spectral peaks range from 700 to 950 nm and show transmittance over 0.6 in all operating wavelengths. We limited the operating wavelength to 950 nm because of the poor efficiency of Si-based image sensor beyond this wavelength. Moreover, the full widths at half maximum (FWHMs) of whole transmission peaks are under 2 nm. This suggests the feasibility of high-resolution spectral imaging using the CMOS-compatible bandpass filter built with nanopost metasurface.

### 2.2 Fabrication and characterization of the device

**Figure 3:**
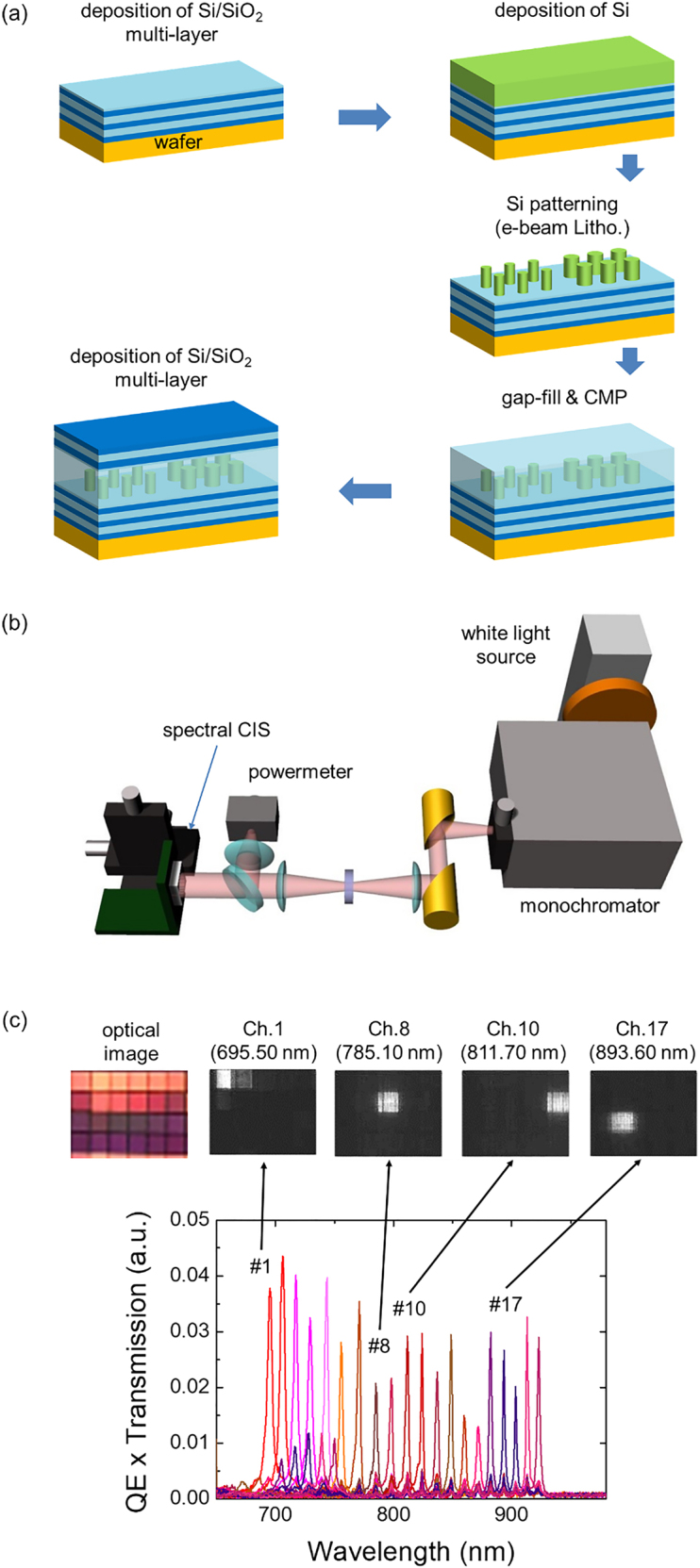
Characterization of meta-spectral imager. (a) Schematic of the whole fabrication processes. (b) Schematic of the spectral imager characterization setup. (c) Measured characteristic spectra of the spectral imager. Top rows are images of some channels taken by the spectral imager. As the incident wavelength increased, the bright channel moves from top-left (the first channel) to bottom-right (the twentieth channel).

We fabricated the metasurface bandpass filter array on the bare CIS wafer (S5K4E8, Samsung) using a standard cleanroom process involving PECVD and dry etching. Figure 3(a) shows whole steps of device fabrication. For the first fabrication step, we deposited multi-layers of Si and SiO_2_ for the bottom dielectric reflector. Electron beam lithography was used to define the nanopost array. Then the nanopost array was formed using inductively coupled plasma reactive ion etching (ICP-RIE), and SiO_2_ was deposited again to fill the gap between nanoposts. Chemical mechanical polishing (CMP) processes were then conducted to flatten the top side of SiO_2_. Lastly, a multi-layer made of Si and SiO_2_ was deposited for the top reflector.

After fabrication, we measured the characteristic spectral response of the fabricated device using the setup shown in Figure 3(b). White light is incident into the monochromator and a narrow spectral band is transmitted through the output slit. The transmitted light is then divided by a beamsplitter. One is incident into the fabricated device and the other is incident into a power-meter to obtain the reference spectrum. By incrementing the wavelength by 0.7 nm from 650 to 1000 nm, we captured spectral images of the fabricated device. Figure 3(c) shows the spectrum of each channel for a channel size of 5.6 μm. The FWHMs of each channel were in the range of 2–5 nm, which is slightly wider than the 2 nm width obtained from RCWA simulations. On the other hand, other sample with large channel size of 28 μm showed FWHMs of about 2 nm. The crosstalk between neighboring channels might have caused the high FWHMs for small channel sizes.

### 2.3 Hyperspectral imaging

**Figure 4:**
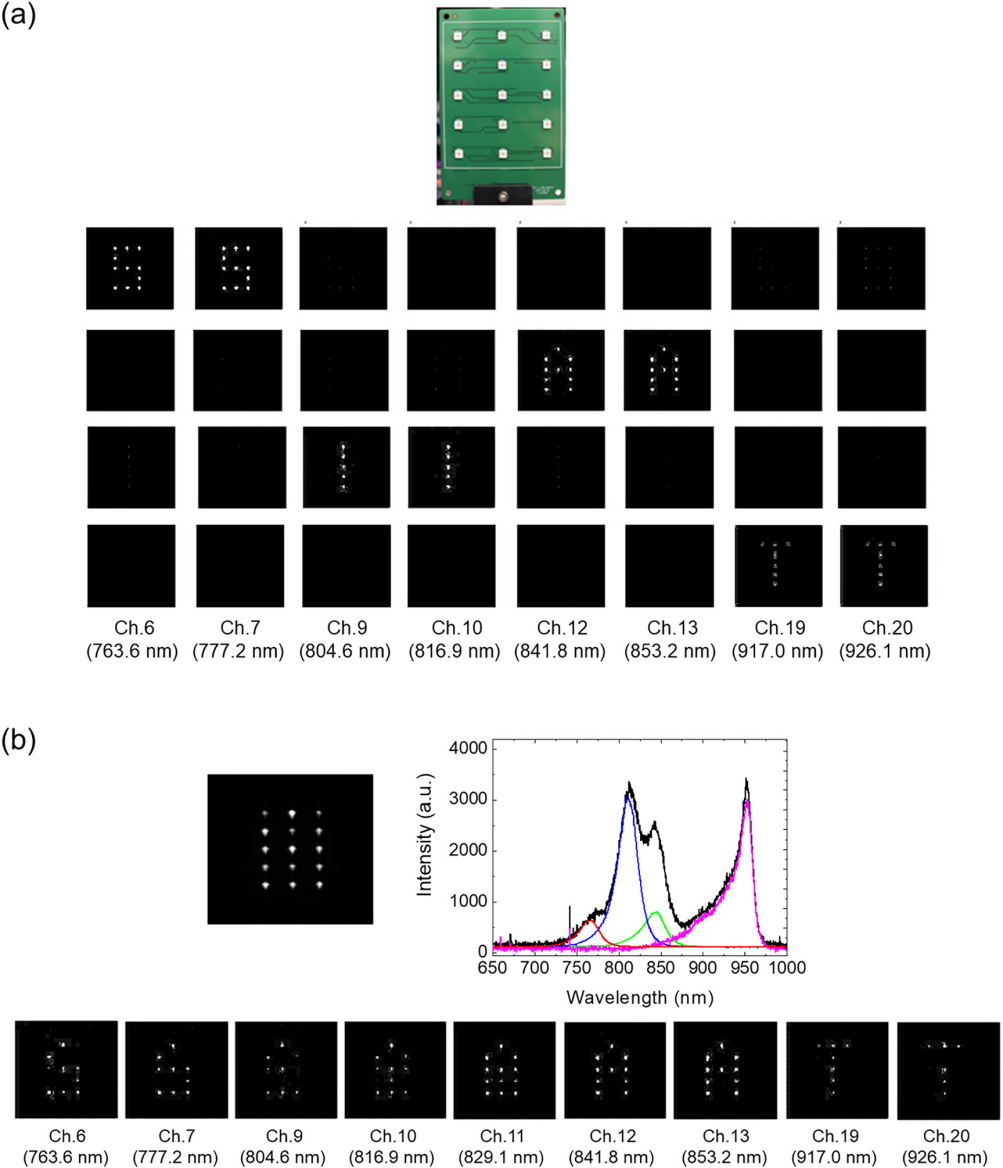
Hyperspectral imaging of the meta-spectral imager. (a) (Top) Photograph of the test panel for spectral imaging. (Bottom) Spectral images measured when only one letter is switched on. Letter is the brightest at the peak wavelength of LED emission. (b) (Top-left) Image of combined letters. (Top-right) Reference spectra of the LED emissions. (Bottom) Spectral images divided into 20 channels.

To demonstrate hyperspectral imaging, we took spectral images of an LED panel comprised of 3 × 5 multi-wavelength LEDs, as shown in the top of Figure 4(a). Each LED emits a combination of multiple wavelengths selected to represent the following upper case letters: 770 nm LEDs for ‘S’, 810 nm for ‘I’, 850 nm for ‘A’, and 950 nm LEDs for ‘T’. With LEDs of each letter turned on, we confirmed the emission wavelength of individual letters from the spectral images, which is shown in the bottom of Figure 4(a) (Supplementary materials). As a proof-of-concept, we took a single-shot image of the panel with all LEDs turned on, which is shown in the top of Figure 4(b). All letters in the image were indistinguishable because all LEDs of the panel were turned on. By dividing this combined image into 20 channels shown in the bottom of Figure 4(b), we revealed the hidden letters of “SAIT”. At channel 11 corresponding to 829.1 nm, “I” and “A” were combined due to the broadband emissions of 810 and 850 nm LEDs. For the longer wavelengths (Ch 12 and 13), we observed that the letter “I” became fainter while letter “A” became clearer. From these experimental results, we confirmed that the meta-spectral imagers show good spectral imaging.

## 3 Conclusions

We presented a compact spectral imager based on metasurface filter array and demonstrated experimentally its hyperspectral-imaging performance. The metasurface bandpass filter array that was directly integrated on CIS produced high resolution hyperspectral images without the need for long optical path and precise alignment of optical components. Because the imager is CMOS-compatible, it is also compatible with conventional mobile image sensors and reliable with portable applications. With further improvements, we expect that the device will serve as a new platform for spectral imaging and open up a new era of applications such as biosensing, food inspection, and mobile health care.

## Supplementary Material

Supplementary Material
