# Supplementary material for: Compact meta-spectral image sensor for mobile applications
Source: Nanophotonics. 2022 Jan 14;11(11):2563–9. doi: 10.1515/nanoph-2021-0706 (PMC11501992; doi:10.1515/nanoph-2021-0706)
Supplement: Supplementary file 1 — Supplementary Material [file j_nanoph-2021-0706_suppl.docx]

**Supplementary Materials**

Compact Meta-Spectral Image Sensor for Mobile Applications

Jaesoong Lee^1†*^, Yeonsang Park^3†^, Hyochul Kim^1^, Young-Zoon Yoon^1^, Woong Ko^2^, Kideock Bae^2^, Jeong-Yub Lee^2^, Hyuck Choo^1^, and Young-Geun Roh^1*^

1Photonic Device Lab., Samsung Advanced Institute of Technology, 130 Samsung-ro, 16678, Suwon, Korea

2Nano Electronics Lab., Samsung Advanced Institute of Technology, 130 Samsung-ro, 16678, Suwon, Korea

3Department of Physics, Chungnam National University, 99 Daehak-ro, 34314, Daejeon, Korea

^†^These authors contributed equally to this work.

*Corresponding authors, e-mail: [jaesoong.lee@samsung.com](mailto:jaesoong.lee@samsung.com) and yg000.roh@samsung.com

**S1. Metasurface bandpass filter**

We designed bandpass filter array covering near-infrared (NIR) range by inserting square nanopost arrays into a resonator with high Q-factor (quality factor). The resonator with high Q-factor was formed by sandwiching a 154 nm thick SiO_2_ layer with two DBRs (Distributed Bragg Reflector). The DBR consisted of 3.5 pairs of a 47.9 nm-thick α-Si layer and a 137.3 nm-thick SiO2, and whole structures were designed CMOS-compatibly. By embedding various nanopost array with different diameter and period in the central SiO_2_ layer, we could change transmission peaks of each bandpass filter by not changing the central thickness of resonator from 700 nm to 950 nm wavelength. This bandpass filter array based on metasurfaces, nanopost array, has several advantages such as tuning ability, wide-angle tolerance for the incidence angle etc. compared to the approach of changing the central thickness of resonator. Especially in the view of the fabrication process, the metasurface-based bandpass filter array requires only one-step lithography and etching process in forming many channels for spectral imaging irrespective of the number of channels. Therefore, this has possibility of fabricating compact spectral image sensors cost-effectively. Table S1 shows the diameter and periodicity of each nanopost corresponding to each channel with the central wavelength. After whole fabrication processes presented in Fig. 3(a) of the main text, we measured spectra of the fabricated spectral image sensor and presented in Fig. 3(c) of the main text. Because of the NIR spectral response of bare CIS shown as a black line in Fig. S1, Figure 3(c) shows spectra that were multiplied by pure transmission of the bandpass filter and the spectral response of bare CIS.

| channel  dimension | 1 | 2 | 3 | 4 | 5 | 6 | 7 | 8 | 9 | 10 |
| --- | --- | --- | --- | --- | --- | --- | --- | --- | --- | --- |
| size (nm) | 90 | 100 | 110 | 120 | 130 | 140 | 150 | 160 | 170 | 180 |
| period (nm) | 170 | 180 | 190 | 200 | 210 | 220 | 230 | 240 | 250 | 260 |
| wavelength (nm) | 706.2 | 717 | 728.8 | 741.4 | 754.4 | 767.8 | 781.4 | 795.2 | 809 | 822.6 |
|  |  |  |  |  |  |  |  |  |  |  |
| channel  dimension | 11 | 12 | 13 | 14 | 15 | 16 | 17 | 18 | 19 | 20 |
| size (nm) | 190 | 200 | 210 | 220 | 230 | 240 | 250 | 260 | 270 | 280 |
| period (nm) | 270 | 280 | 290 | 300 | 310 | 320 | 330 | 340 | 350 | 360 |
| wavelength (nm) | 836 | 849 | 861.6 | 873.8 | 885.6 | 897.2 | 908.4 | 919.2 | 929.8 | 940.2 |

**Table S1.** Sizes, periods, simulated peak wavelengths of nanoposts for 20 channels.


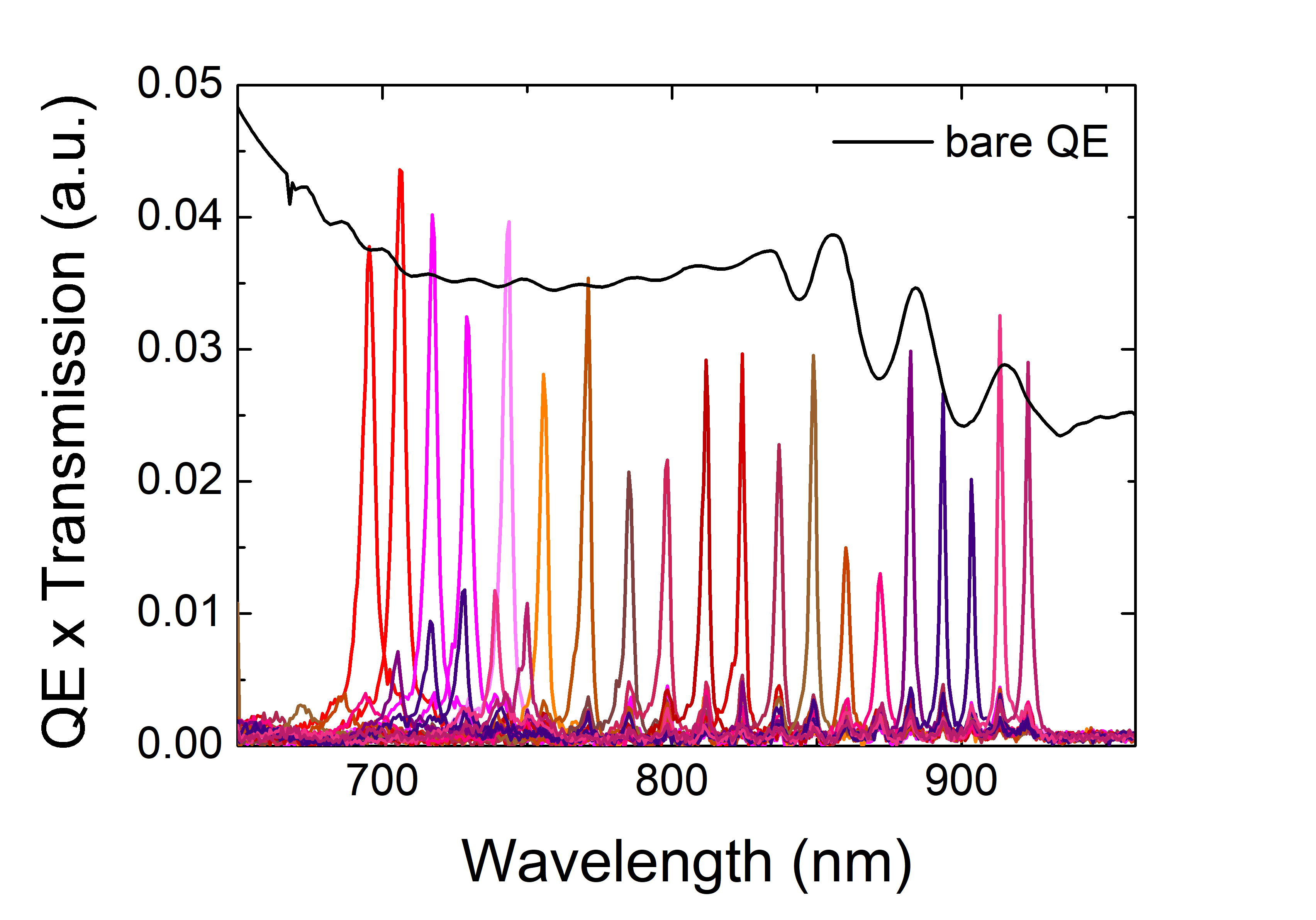


**Figure S1**. Measured QE spectrum of the bare CIS and measured spectra of the fabricated spectral CIS.

**S2. Hyperspectral imaging and their spectra**

To make it sure of ability of the fabricated meta-spectral CIS, we measured hyperspectral imaging experiment using letter panel with different LEDs. Figure S2(a) shows images of our spectral imaging experiment. As commented in the maintext, the fabricated spectral CIS was operated by Samsung evaluation kit called as “Simmian” board. Meta-spectral CIS was attached to the board and the board was operated by a labtop computer. In the front of the board, standard imaging camera was combined with the board and we took spectral images. The “SAIT” letter LED panel was switched-on sequentially, and presented each letter with different wavelength emission. From images taken by the meta-spectral CIS, we could divide images with 20 channels shown in Figure 4(a) of the main text, and also obtain spectra of each LED emission showing each letter. Figure S2(b) shows obtained spectra of each LED emission and compared with the reference spectrum measured by a conventional spectrometer. From Fig. 2(b), we could confirm that our meta-spectral CIS has spectral ability of discriminating combined spectra into each channel.


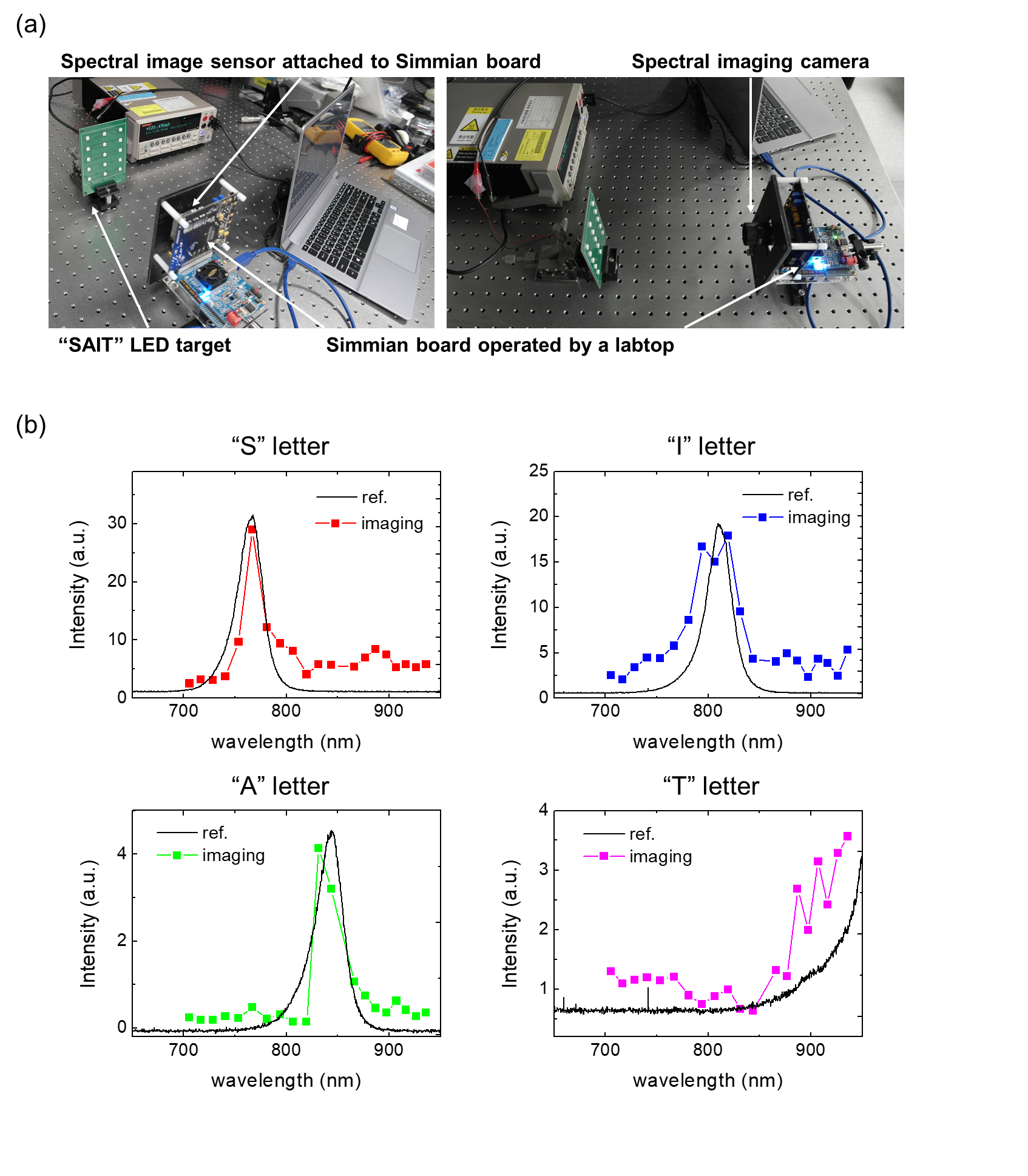


**Figure S2.** (a) Image of hyperspectral imaging setup. (b) Spectra of 4-letter target obtained from spectral images taken by our meta-spectral CIS.
